# The Influence of Sociocultural Determinants on the Number of Diagnosed Chronic Illness Reported by Indigenous Peoples in Canada and the United States During SARS-CoV2

**DOI:** 10.1177/21501319251354833

**Published:** 2025-07-04

**Authors:** Mary G. Jessome, Kimberly R. Huyser, Katherine A. Collins, Tom Einhorn, Tamara Chavez, Nicole Dawydiuk, Michelle Johnson-Jennings

**Affiliations:** 1 Department of Sociology, University of British Columbia, Vancouver, BC, Canada; 2 Department of Psychology and Health Studies, University of Saskatchewan, Saskatoon, SK, Canada; 3 School of Social Work, University of Washington, Seattle, WA, USA

**Keywords:** chronic illness, Indigenous health, COVID-19, culture, land

## Abstract

**Objectives:**

To determine the influence of cultural, including land-based, factors on the reported number of diagnosed chronic illnesses among Indigenous individuals living in Canada and the United States during SARS-CoV-2 (COVID-19).

**Methods:**

557 Indigenous individuals completed the *Hearing Indigenous Voices* survey (HIS) in 2021. Data from HIS respondents living with at least one chronic illness was used to conduct a Poisson regression. This equation estimated the effect of change in ancestral land use, participation in cultural activities, and demographic characteristics on the number of diagnosed chronic illnesses reported by Indigenous individuals.

**Results:**

Results demonstrate that the number of chronic illnesses reported by Indigenous individuals during COVID-19 was positively correlated with 2 cultural factors. The first is spending a different amount of time on ancestral territories compared to before the pandemic (*P* < .01). Participating in beading, traditional arts and crafts, or Indigenous storytelling (*P* < .001) is the second. However, this relationship was moderated by age (*P* < .01) and socio-economic status (*P* < .001), with positive and negative associations respectively found for each demographic factor.

**Discussion:**

Cultural practices, including accessing ancestral territories, often positively contribute to Indigenous Peoples’ health. The increased number of diagnosed chronic illnesses among respondents who participate in cultural activities suggests that those living with chronic illness may not gain the same benefits from culture during pandemics because of the multitude of barriers they face during emergencies.

## Introduction

The Indigenous Peoples of Turtle Island, a term referring to the geographic region known as Canada and the United States, come from culturally rich and healthy communities. However, they tend to bear the disproportionate burden of chronic illnesses like cancer, diabetes, cardiovascular disease, HIV/AIDS and chronic respiratory disease.^1-3^ Though progress has been made, racial disparities in the rate of chronic illness have persisted for decades, even increasing in recent years, despite well-intentioned efforts from health researchers and practitioners to understand and address the factors that prevent Indigenous Peoples from being well.^4,5^ The continuance of chronic conditions is a multi-factorial issue associated with the stress of living with the intergenerational trauma caused by colonization,^1-3^ experiences of anti-Indigenous racism within healthcare settings,^6-8^ the continuation of health behaviours that contribute to disease development,^9,10^ decreased capacity within the healthcare system to provide care for patients with complex illnesses or a lack of culturally informed programmes to address the health concerns of Indigenous Peoples.^11-13^

The social vulnerabilities that Indigenous Peoples living with chronic illness face became apparent during SARS-CoV-2 (COVID-19). The factors outlined above converged with a greater likelihood of contracting and experiencing complications from an infectious disease, as well as encountering emergency-specific public health policies as barriers to accessing routine care.^14,15^ Data from early in the COVID-19 pandemic highlight how unsupported Indigenous Peoples living with chronic illness were during this period. Of those living off-reserve, 21% reported their healthcare needs being unmet. This lack of care was associated with racial disparities in reported rates of chronic illness. 15% of off-reserve First Nations and 16% of Métis reported living with 2 chronic illnesses in 2021.^1^ This compared to 12% of the non-Indigenous population. The gap was even greater for 3 or more conditions, with respective rates of 14% and 8% reported by First Nations and non-Indigenous individuals.^1^

Calls for culturally informed practices like land-based healing or traditional arts and crafts emerged in parallel to Indigenous Peoples’ healthcare needs going unmet during COVID-19. Elders and community leaders viewed these practices as a critical component of living with and healing from the polycrises that arose during the pandemic.^16-18^ Community support for culturally informed public health practices during the pandemic reflected a growing desire to reclaim culture and land as a source of holistic wellbeing.^19,20^ Despite their importance to Indigenous communities, public health organizations and officials frequently neglected requests, opting for biomedical-style interventions, despite their poor efficacy in addressing illness among Indigenous Peoples.^21^ Given the obstruction to accessing health services and the call from community leaders to draw on traditional practices, the COVID-19 pandemic presents a unique opportunity to explore the influence of cultural, including land-based, factors on the lives of Indigenous Peoples during health emergencies. Our team of Indigenous and allied researchers explore this relationship, using data from the 2021 *Hearing Indigenous Voices* survey (HIS) to examine the association between cultural, including ancestral land use, and the number of diagnosed chronic illnesses reported by Indigenous Peoples living in Canada and the United States during COVID-19. This correlational study deepens our understanding of the relationship between culture and chronic illness by investigating how this association functions during health emergencies. Although culture often plays an important role in keeping Indigenous Peoples healthy, the barriers experienced by Indigenous Peoples living with chronic illness during pandemics likely make participation in cultural activities less accessible, altering how culture influences health.

## Methods

Our reporting of the HIS follows the *Checklist for Reporting Results of Internet E-Surveys* (CHERRIES) method.^22^ The HIS was a cross-sectional survey designed to study the influence of COVID-19 on Indigenous Peoples residing in Canada and the United States, including 67 questions about COVID-19-related attitudes and behaviours. These questions measure areas such as participants’ demographic characteristics, vaccine uptake, participation in cultural and land-based activities during COVID-19, attitudes towards the pandemic and its associated public health measures, as well as lifestyle changes experienced during COVID-19. Data collection occurred in October and November 2021. Participants were recruited from a panel maintained by the staff of Qualtrics. Self-identified Indigenous members of the panel were sent an invitation by email to complete the survey.

After providing consent, potential respondents were asked qualifying questions. Only those who self-identified as Indigenous and who lived in Canada or the United States were able to proceed to the remainder of the survey. Those who did not meet these criteria ended the questionnaire. Qualtrics staff anonymized all response data and compensated participants with a small financial reward before sending the data to our research team. The final data indicated that 557 of 1760 respondents completed the survey, marking a 32% completion rate. The 557 respondents constitute a non-probability sample. Ethics approval for this study was provided by a university research ethics board.

In this article, we use HIS data to estimate the effect of land use, cultural activities and demographic characteristics on the number of diagnosed chronic illnesses reported by participants. Our dependent variable is a count of diagnosed chronic conditions. See Appendix A for the full list of chronic conditions included in the survey. The number of conditions reported by each respondent was then summed and is represented in our model as a discrete, non-negative integer dependent variable ranging from 0 to 10.

Our model was developed through a systematic, iterative process. We started by creating a comprehensive set of theoretically relevant variables included in HIS data. Given that our dependent variable represents count data and follows a Poisson distribution, we employed a Poisson regression model. As the standard analytical approach to modelling count data, Poisson regressions appropriately account for the non-normal distribution and heteroscedasticity inherent in count data. We assessed model fit using BIC and log likelihood comparisons, examining for potential over-dispersion that would decrease the appropriateness of the model to our data. Using both measures helped us balance explanatory power and parsimony. In our examination, we found minor over-dispersion in our dependent variable.

As a robustness check, we fitted identical Poisson and negative binomial models, which are recommended for over-dispersed count data, at each stage. Although not reported here, negative binomial models produced substantively similar results, with coefficients matching to 3 decimal places. This confirms that the minor over-dispersion detected in our data did not meaningfully inform our estimates and suggests that the effect sizes reported in this paper are robust to the choice of model. All models were fitted using R. We systematically eliminated variables that did not significantly improve model fit through this approach, resulting in a more efficient final model. While alternative models with additional demographic and cultural variables were tested, the model reported in this paper performed better on our selected criteria despite its more parsimonious structure. This evidence-based variable selection process ultimately resulted in a model that maximizes explanatory value while adhering to principles of statistical efficiency.

**Figure 1.**
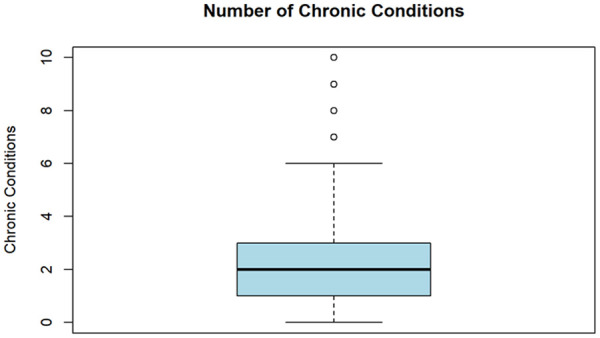
Distribution of the number of chronic conditions.

Figure 1 shows the distribution of our dependent variable. The measure ranges from 0 to 10, with 10 being the maximum co-occurring chronic conditions reported by respondents. The mean number of chronic illnesses was 2.08, and the median was 2. Overall, 77% of the sample reported being diagnosed with at least one of the chronic illnesses listed in Appendix A. Additionally, Table 1 shows the prevalence of chronic illnesses grouped thematically. Reported by 50% of the sample, the most common grouping was mental health conditions, which includes diagnoses of anxiety, depression, post-traumatic stress disorder and other mental health conditions. This was followed by cardiovascular and metabolic conditions, respiratory conditions, organ-specific conditions, and immune system and infectious diseases. In Canada alone, rates of chronic illness reported in our convenience sample are about 30% higher than the national average among First Nations, Métis and Inuit people. In combination with the barriers to care Indigenous Peoples living with chronic illness faced during COVID-19, the over-representation of chronic illness means that findings should be interpreted and applied with caution.

**Table 1.** Prevalence of Chronic Illnesses.

Group	Percentage (N)
Mental health conditions	50.63 (282)
Cardiovascular and metabolic conditions	39.5 (220)
Respiratory conditions	22.62 (126)
Organ-specific conditions	11.67 (65)
Immune system and infectious diseases	10.05 (56)
None of the above	22.8 (127)

Three independent variables were selected to measure the influence of cultural factors on Indigenous health during COVID-19. The first is the change in the amount of time spent on ancestral lands. At the time of completing the survey, the HIS asked respondents how much time they spend on land compared to before the COVID-19 pandemic. Those participating were provided with 6 possible response options: A lot more, somewhat more, the same as before, somewhat less, a lot less, and not applicable. Respondents who answered ‘not applicable’ (n = 101) were removed from the analysis. The remaining respondents (n = 456) were recorded into a variable with 2 levels. Respondents reporting that they either spent more or less time on traditional lands compared to before COVID-19 were grouped into the ‘change’ category, representing participants whose connection to the land had been affected by the pandemic. Respondents answering ‘the same as before’ were recorded into the ‘no change’ group. We interpreted this variable as recording whether the pandemic shifted respondents’ connection to the land. Respondents in the ‘change’ category either increased or decreased the time spent on land during the pandemic, whereas the ‘no change’ category includes respondents for whom the pandemic made no difference to their time spent on land. This latter category serves as the reference category in the analysis presented below.

The next 2 independent variables were cultural indices. Each index captures respondents’ participation in different cultural practices. The first focuses on land-related practices like hunting, fishing or gathering medicine. The second focuses on Indigenous art, beadwork and telling Indigenous stories. Finally, given the overrepresentation of chronic illness among certain populations (i.e. women, older populations or those who are low-income), we selected 4 control variables. These include age, as was reported in years; gender, which was reported using self-identification, with male used as the reference category; socio-economic status, which was operationalized as having enough money to meet one’s needs, with having ‘a little’ or ‘not at all’ used as the reference category; and, community belonging and support, which was operationalized as the degree to which respondents feel they belong to, and are supported by, their local community.

## Results

Our study fitted a Poisson model to evaluate the relationship between change in time spent on ancestral lands and cultural indices on the number of diagnosed chronic illnesses reported by participants. Poisson models output coefficients on a log scale. As such, it is difficult to directly interpret the influence of changes in predictor variables on the outcome without first converting coefficients to more intuitive measures. In Table 2, we report exponentiated coefficients, which are easier to interpret. They represent change in the ratio of expected counts of reported chronic conditions between comparison groups. To reflect the cross-sectional nature of our study and to avoid confusion with longitudinal incidence measures, we report these as Prevalence Rate Ratios (PRRs).

**Table 2.** Poisson Regression Results.

Variable	Model Results		
	PRR	95% CI	*P*-value
Time on land			
No change	—	—	
Change	1.25	1.08, 1.44	.002
Age	1.01	1.00, 1.01	<.001
Gender			
Male	—	—	
Female or other	1.14	0.98, 1.32	.10
Enough money			
Not at all or a little	—	—	
Moderately	0.85	0.72, 1.01	.065
Mostly or completely	0.67	0.56, 0.79	<.001
Community belonging	0.99	0.98, 1.01	.3
Land-based activities			
None	—	—	
At least one	0.89	0.74, 1.10	.3
Traditional Indigenous Arts and crafts			
None	—	—	
At least one	1.32	1.14, 1.53	<.001

Abbreviations: CI, confidence interval; IRR, incidence rate ratio.

Number of observations = 380; AIC = 1446; Log Likelihood = −714.

Results show that respondents who reported a change in their relationship with ancestral and traditional lands over the pandemic are projected to report 25% more diagnosed chronic conditions compared to respondents who reported no change (*P* < .01). Additionally, engaging in traditional Indigenous arts and crafts corresponds to an increase of 32% in the number of reported chronic conditions (*P* < .001). The land-based index does not make a statistically significant contribution. As for demographic variables, each increase in age corresponds to a 1% increase in the number of reported chronic conditions (*P* < .01), whereas having enough money to meet one’s needs is associated with a 33% decrease in the number of reported chronic conditions compared to not having enough money to meet one’s needs (*P* < .001). There is no statistically significant contribution associated with respondents’ gender.

## Discussion

Having deepened existing health disparities, the COVID-19 pandemic emphasized the urgent need to support Indigenous Peoples. Rates of chronic illness rose as necessary health services were restricted by public health measures.^14,15^ Indigenous Elders and community leaders called for individuals to draw on cultural traditions like returning to the land or participating in beading campaigns in response to emerging issues. This analysis acknowledges the revival of traditional practices, examining the association between cultural factors and the number of diagnosed chronic illnesses reported by Indigenous Peoples living in Canada and the United States. Our findings suggest that changes in land use and participation in cultural activities are associated with an overall increase in the number of chronic illnesses reported by respondents.

Culture, including land, is generally beneficial to Indigenous health, making our findings counterintuitive. However, traditional practices, particularly land-based activities that promote healing, are often used by individuals with complex health conditions that cannot be easily treated or cured by the healthcare system.^16,23,24^ The positive correlation between land, culture and chronic illness found in our analysis may be indicative of this tendency. Alternatively, only beading, doing other traditional arts and crafts, or sharing Indigenous stories are the only activities significantly associated with the reported number of diagnosed chronic illnesses. While our correlational data lacks the measures necessary to determine why these specific activities were associated with chronic illness during the COVID-19 pandemic, these activities could reflect the need to make cultural practices accessible. Where changes to land use may reflect the barriers those experiencing less stable health faced during COVID-19, participating in traditional arts and crafts and Indigenous storytelling could have been easier to access. Further research will be needed to clarify our findings.

The influence of land and culture is moderated by various other factors. Results indicate that age is correlated with a 1% increase in the number of reported chronic conditions (*P* < .01). This aligns with the broader understanding of chronic illness, which finds that people experience more chronic health conditions as they age.^25,26^ Socio-economic status has a much stronger influence on respondent outcomes. Having enough money to meet one’s needs correlates with a 33% decrease in the number of chronic conditions reported by respondents. Socio-economic status, like age, is a well-documented determinant of health. This is especially true for Indigenous Peoples, as finances are reported as one of the main obstacles to accessing ancestral lands, affording the supplies and materials necessary to participate in traditional practices, or accessing culturally informed care.^15,26,27^

## Conclusions

The efficacy of land-based and other culturally informed practices in addressing Indigenous health concerns is well-documented by researchers, providing a scientific basis for the wisdom held by Indigenous communities. Health emergencies are predicted to increase in the coming years. As such, it will be important to collect representative, longitudinal data. Doing so will allow researchers to better understand the association between culture and chronic illness during pandemics, and for health practitioners to provide equitable and evidence-based care to Indigenous Peoples.

## Appendix A: List of Chronic Conditions

Question 25: Have you ever been told by a doctor, nurse or medical provider that you have: (Select all that apply)

- High blood pressure or hypertension
- Diabetes (not including pre-diabetes)
- Pre-diabetes
- Cardiovascular disease or heart disease (including blocked or hardening of the arteries, angina, heart attack, stroke or mini stroke)
- Congestive heart failure (including weakened heart muscle or a leaky valve)
- Lung disease (like asthma, exercise-induced asthma, chronic bronchitis, emphysema, COPD)
- Cancer that you are getting treatment for now
- Cancer (not currently getting treatment)
- Autoimmune disease (like lupus, rheumatoid arthritis, psoriasis)
- Kidney disease, including weak or failing kidneys (DO NOT include kidney stones or problems with urinating)
- HIV/AIDS
- Hepatitis B or Hepatitis C
- Sleep disorders/apnoea
- Low immunity/suppressed immune system (or any medication that decreases your immunity, such as for transplant or an immune disease).
- Chronic liver disease (like fatty liver, cirrhosis)
- Reproductive system disease (infertility, genetic disease, congenital abnormalities, delayed puberty, precocious puberty, dysmenorrhea)
- Anxiety
- Depression
- Post-Traumatic Stress Disorder
- Other mental health condition (please specify): ________________________
